# Supplementary material for: Proteasome inhibition-enhanced fracture repair is associated with increased mesenchymal progenitor cells in mice
Source: PLoS One. 2022 Feb 25;17(2):e0263839. doi: 10.1371/journal.pone.0263839 (PMC8880819; doi:10.1371/journal.pone.0263839)
Supplement: S1 Table — (PPT) [file pone.0263839.s001.ppt]

## Slide 1
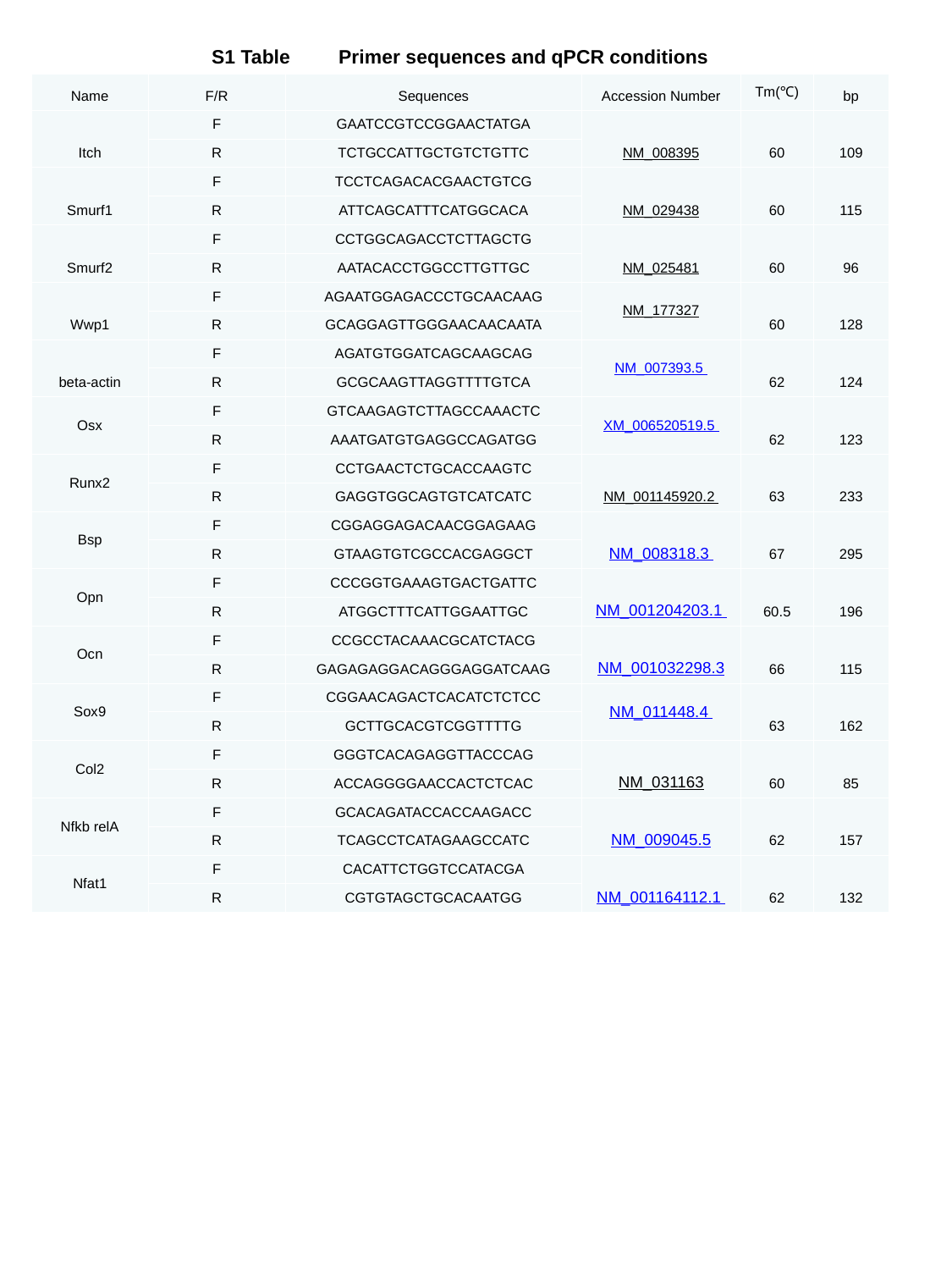

S1 Table 	Primer sequences and qPCR conditions
| Name | F/R | Sequences | Accession Number | Tm(℃) | bp |
| --- | --- | --- | --- | --- | --- |
| Itch | F | GAATCCGTCCGGAACTATGA | NM\_008395 | 60 | 109 |
| | R | TCTGCCATTGCTGTCTGTTC | | | |
| Smurf1 | F | TCCTCAGACACGAACTGTCG | NM\_029438 | 60 | 115 |
| | R | ATTCAGCATTTCATGGCACA | | | |
| Smurf2 | F | CCTGGCAGACCTCTTAGCTG | NM\_025481 | 60 | 96 |
| | R | AATACACCTGGCCTTGTTGC | | | |
| Wwp1 | F | AGAATGGAGACCCTGCAACAAG | NM\_177327 | 60 | 128 |
| | R | GCAGGAGTTGGGAACAACAATA | | | |
| beta-actin | F | AGATGTGGATCAGCAAGCAG | NM\_007393.5 | 62 | 124 |
| | R | GCGCAAGTTAGGTTTTGTCA | | | |
| Osx | F | GTCAAGAGTCTTAGCCAAACTC | XM\_006520519.5 | 62 | 123 |
| | R | AAATGATGTGAGGCCAGATGG | | | |
| Runx2 | F | CCTGAACTCTGCACCAAGTC | NM\_001145920.2 | 63 | 233 |
| | R | GAGGTGGCAGTGTCATCATC | | | |
| Bsp | F | CGGAGGAGACAACGGAGAAG | NM\_008318.3 | 67 | 295 |
| | R | GTAAGTGTCGCCACGAGGCT | | | |
| Opn | F | CCCGGTGAAAGTGACTGATTC | NM\_001204203.1 | 60.5 | 196 |
| | R | ATGGCTTTCATTGGAATTGC | | | |
| Ocn | F | CCGCCTACAAACGCATCTACG | NM\_001032298.3 | 66 | 115 |
| | R | GAGAGAGGACAGGGAGGATCAAG | | | |
| Sox9 | F | CGGAACAGACTCACATCTCTCC | NM\_011448.4 | 63 | 162 |
| | R | GCTTGCACGTCGGTTTTG | | | |
| Col2 | F | GGGTCACAGAGGTTACCCAG | NM\_031163 | 60 | 85 |
| | R | ACCAGGGGAACCACTCTCAC | | | |
| Nfkb relA | F | GCACAGATACCACCAAGACC | NM\_009045.5 | 62 | 157 |
| | R | TCAGCCTCATAGAAGCCATC | | | |
| Nfat1 | F | CACATTCTGGTCCATACGA | NM\_001164112.1 | 62 | 132 |
| | R | CGTGTAGCTGCACAATGG | | | |
