# Supplementary material for: Proteasome inhibition-enhanced fracture repair is associated with increased mesenchymal progenitor cells in mice
Source: PLoS One. 2022 Feb 25;17(2):e0263839. doi: 10.1371/journal.pone.0263839 (PMC8880819; doi:10.1371/journal.pone.0263839)
Supplement: S1 Fig — C57/B6 WT mice and Itch knockout out mice received open tibial fracture surgery and were sacrificed at 10, 14, 21, and 28 days post fracture. Fractured tibiae examined by micro-CT. (A) Representative micro-CT images showing fracture callus. (B) Callus volume analyzed from micro-CT images. N = 3-5/time points. (PPT) [file pone.0263839.s002.ppt]

## Slide 1
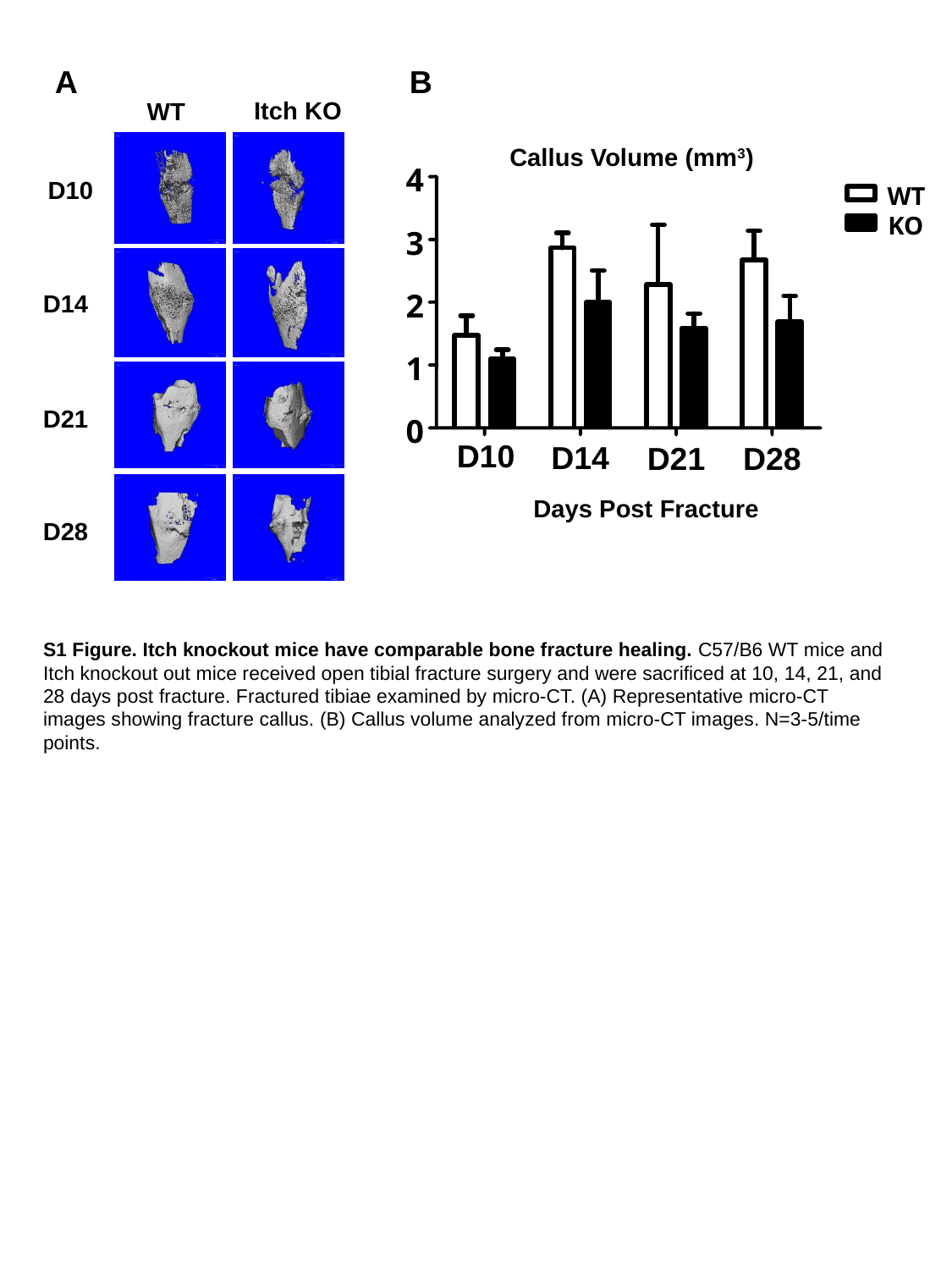

A
B
Itch KO
WT
Callus Volume (mm3)
4
WT
KO
3
2
1
0
D10
D14
D21
D28
D10
D14
D21
Days Post Fracture
D28
S1 Figure. Itch knockout mice have comparable bone fracture healing. C57/B6 WT mice and Itch knockout out mice received open tibial fracture surgery and were sacrificed at 10, 14, 21, and 28 days post fracture. Fractured tibiae examined by micro-CT. (A) Representative micro-CT images showing fracture callus. (B) Callus volume analyzed from micro-CT images. N=3-5/time points.
