# Supplementary material for: Proteasome inhibition-enhanced fracture repair is associated with increased mesenchymal progenitor cells in mice
Source: PLoS One. 2022 Feb 25;17(2):e0263839. doi: 10.1371/journal.pone.0263839 (PMC8880819; doi:10.1371/journal.pone.0263839)
Supplement: S1 File — (PPT) [file pone.0263839.s003.ppt]

## Slide 1
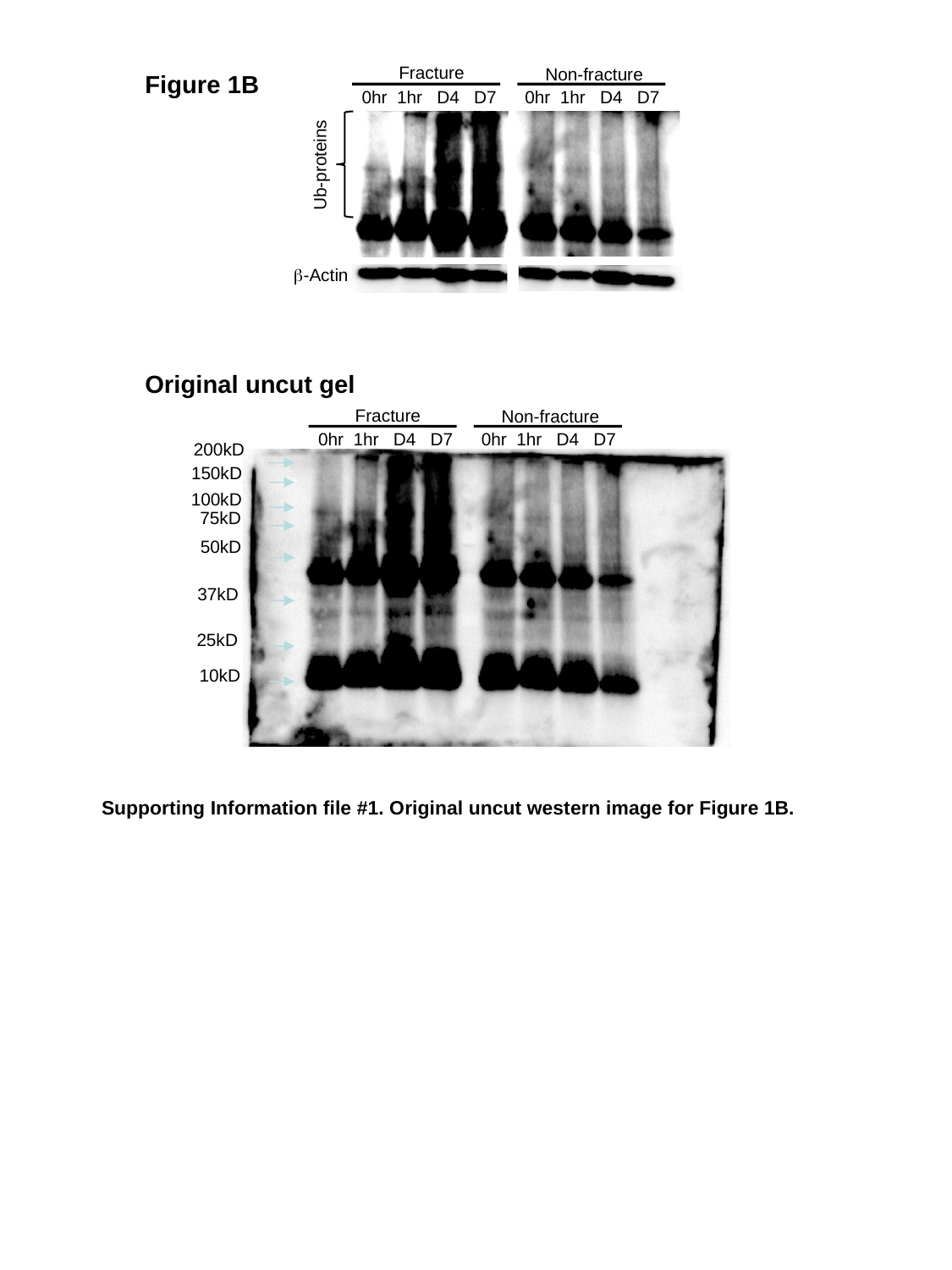

Fracture
Non-fracture
Figure 1B
0hr 1hr D4 D7
0hr 1hr D4 D7
Ub-proteins
β-Actin
Original uncut gel
Fracture
Non-fracture
0hr 1hr D4 D7
0hr 1hr D4 D7
 200kD
 150kD
 100kD
75kD
50kD
37kD
25kD
10kD
Supporting Information file #1. Original uncut western image for Figure 1B.

## Slide 2
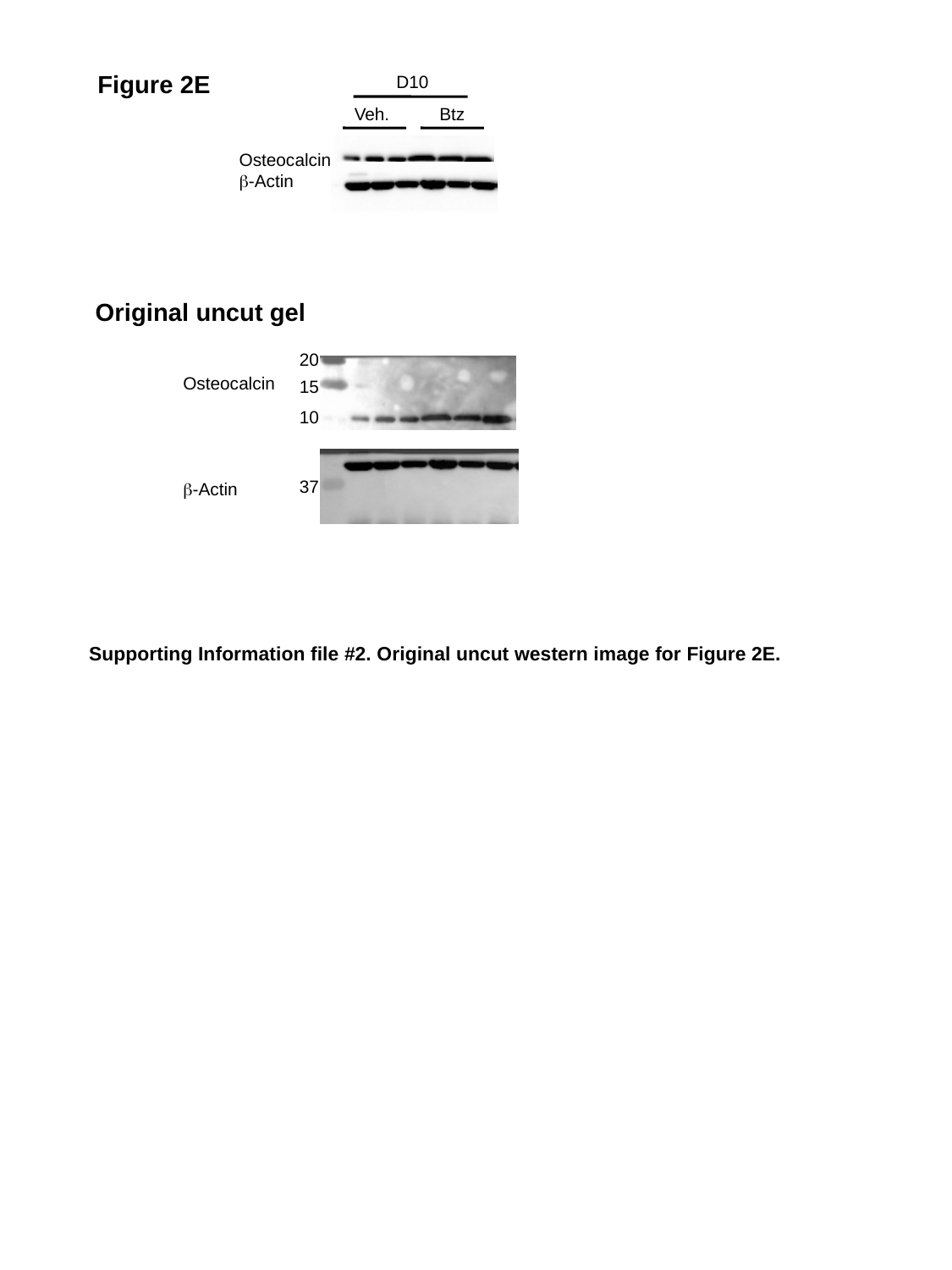

Figure 2E
D10
Veh.
Btz
Osteocalcin
β-Actin
Original uncut gel
20
Osteocalcin
β-Actin
15
10
37
Supporting Information file #2. Original uncut western image for Figure 2E.

## Slide 3
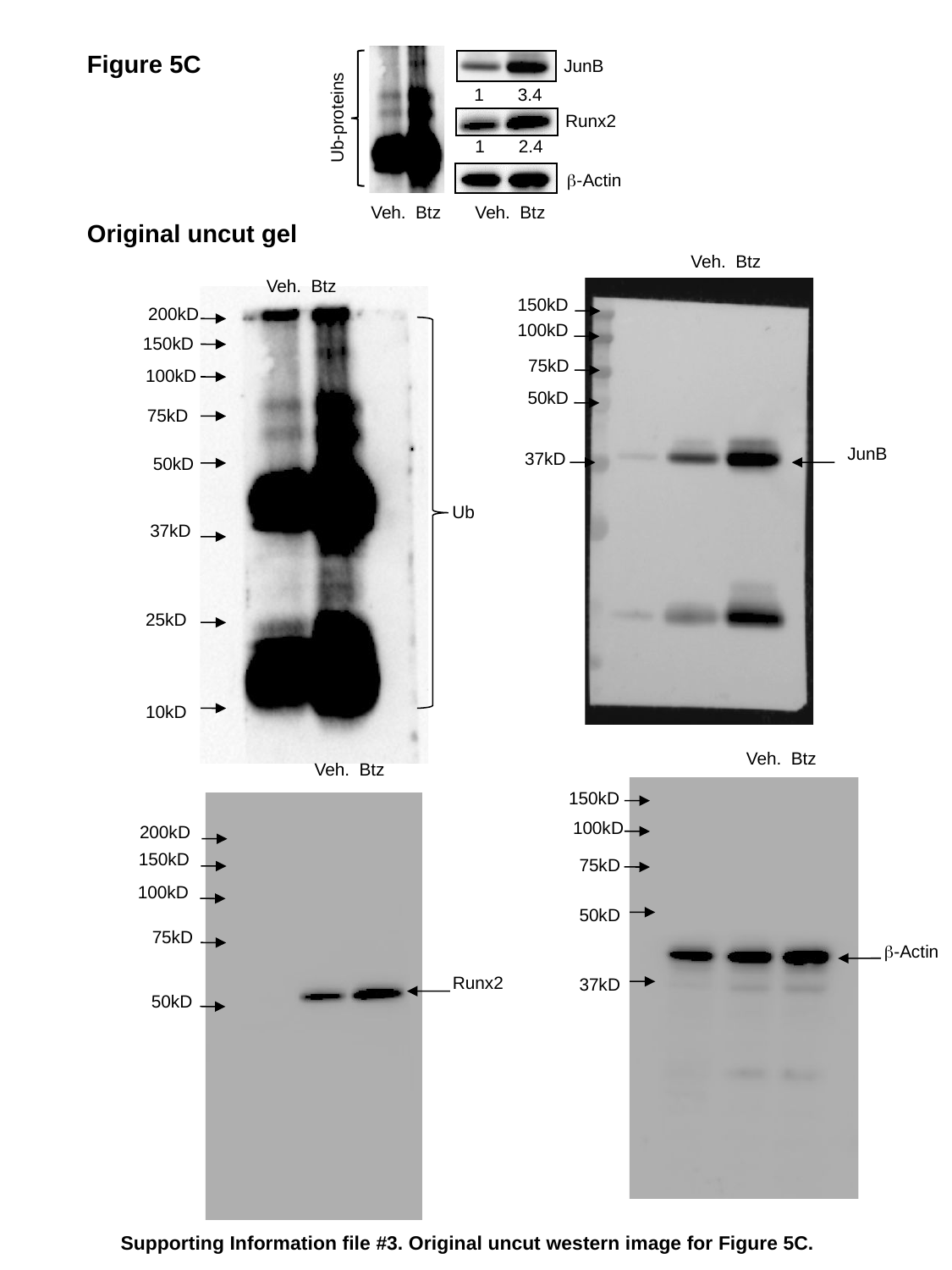

Figure 5C
JunB
1
3.4
Ub-proteins
Runx2
1
2.4
β-Actin
Veh. Btz Veh. Btz
Original uncut gel
Veh. Btz
Veh. Btz
150kD
200kD
100kD
150kD
75kD
100kD
50kD
75kD
JunB
37kD
50kD
Ub
37kD
25kD
10kD
Veh. Btz
150kD
100kD
75kD
50kD
β-Actin
37kD
Veh. Btz
200kD
150kD
100kD
75kD
Runx2
50kD
Supporting Information file #3. Original uncut western image for Figure 5C.

## Slide 4
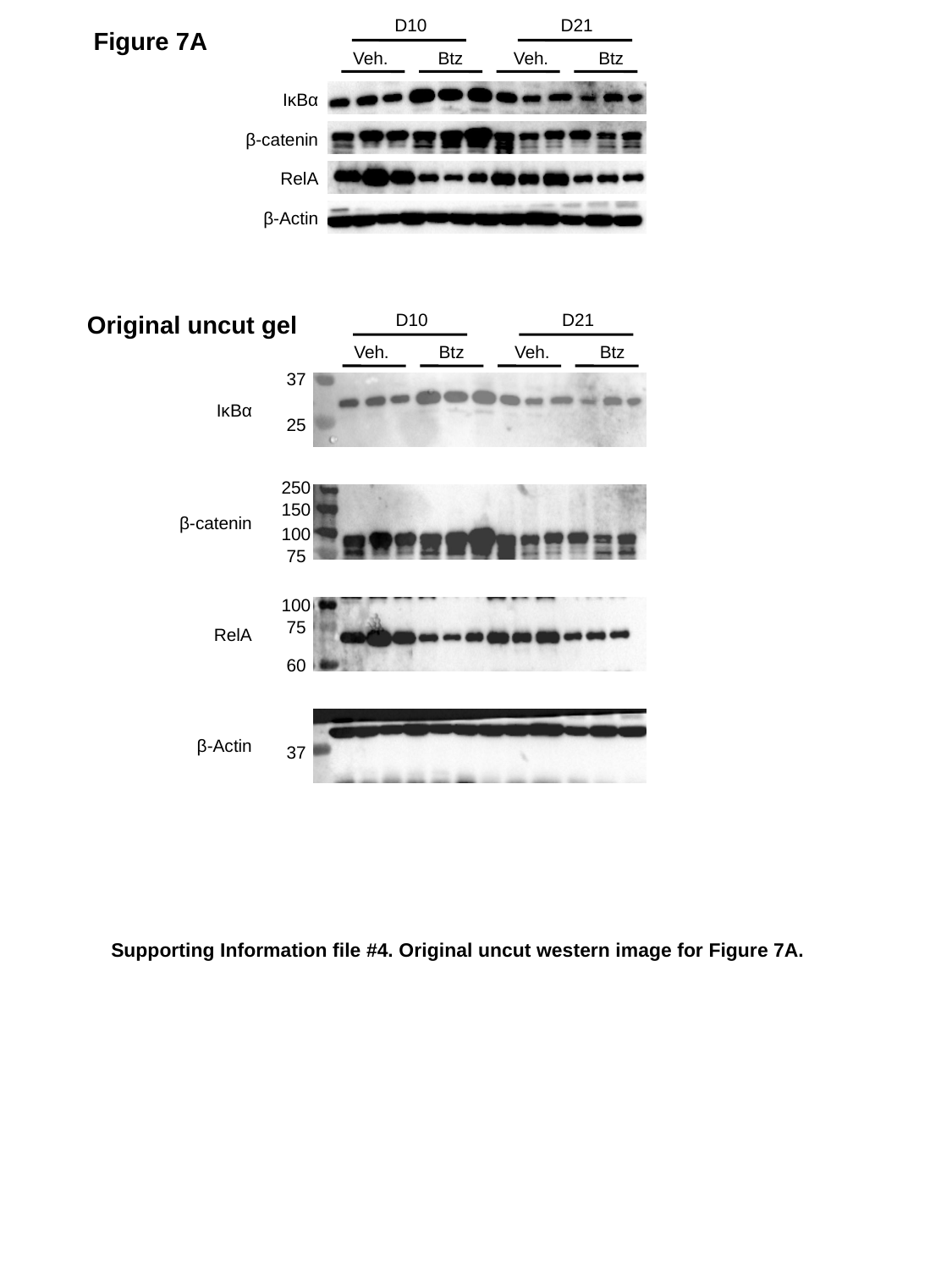

D10
D21
Figure 7A
Veh.
Btz
Veh.
Btz
IκBα
β-catenin
RelA
β-Actin
D10
D21
Original uncut gel
Veh.
Btz
Veh.
Btz
37
IκBα
25
250
150
β-catenin
100
75
100
75
RelA
60
β-Actin
37
Supporting Information file #4. Original uncut western image for Figure 7A.
